# Emotional body postures affect inhibitory control only when task-relevant

**DOI:** 10.3389/fpsyg.2022.1035328

**Published:** 2022-11-03

**Authors:** Marta Calbi, Martina Montalti, Carlotta Pederzani, Edoardo Arcuri, Maria Alessandra Umiltà, Vittorio Gallese, Giovanni Mirabella

**Affiliations:** ^1^Department of Medicine and Surgery, Unit of Neuroscience, University of Parma, Parma, Italy; ^2^Lab Neuroscience & Humanities, University of Parma, Parma, Italy; ^3^Department of Philosophy, State University of Milan, Milan, Italy; ^4^Department of Clinical and Experimental Sciences, University of Brescia, Brescia, Italy; ^5^Department of Food and Drug Sciences, University of Parma, Parma, Italy; ^6^IRCCS Neuromed, Pozzilli, Italy

**Keywords:** emotional body postures, task-relevance, emotional body language, Go/No-go task, inhibitory control

## Abstract

A classical theoretical frame to interpret motor reactions to emotional stimuli is that such stimuli, particularly those threat-related, are processed preferentially, i.e., they are capable of capturing and grabbing attention automatically. Research has recently challenged this view, showing that the task relevance of emotional stimuli is crucial to having a reliable behavioral effect. Such evidence indicated that emotional facial expressions do not automatically influence motor responses in healthy young adults, but they do so only when intrinsically pertinent to the ongoing subject’s goals. Given the theoretical relevance of these findings, it is essential to assess their generalizability to different, socially relevant emotional stimuli such as emotional body postures. To address this issue, we compared the performance of 36 right-handed participants in two different versions of a Go/No-go task. In the Emotional Discrimination task, participants were required to withhold their responses at the display of emotional body postures (fearful or happy) and to move at the presentation of neutral postures. Differently, in the control task, the same images were shown, but participants had to respond according to the color of the actor/actress’ t-shirt, disregarding the emotional content. Results showed that participants made more commission errors (instances in which they moved even though the No-go signal was presented) for happy than fearful body postures in the Emotional Discrimination task. However, this difference disappeared in the control task. Such evidence indicates that, like facial emotion, emotional body expressions do not influence motor control automatically, but only when they are task-relevant.

## Introduction

Humans stand out in their ability to make sense of others’ behavior and establish appropriate social bonds with others. Items laden with affective significance have crucial importance in decision-making as they impact the way cognitive functions operate, enhancing or impairing behavioral performance (Pessoa, 2009). One of the most popular models used to interpret behavioral reactions to emotional stimuli is the motivational model (Bradley et al., 2001; Lang and Bradley, 2010). This model predicts that positive valenced stimuli would activate the appetitive system, while negative valenced stimuli would activate the defensive system. A pillar of the model is the idea that emotional stimuli, especially threatening ones, would be capable of automatically grabbing selective attention, prioritizing their processing, and eliciting behavioral responses independently from the subject’s current goals (Lang et al., 2000; Vuilleumier, 2005). However, the empirical evidence about how emotional stimuli influence motor control is contradictory and does not provide consistent support to this hypothesis (for a literature survey, see Mancini et al., 2020, 2022; Mirabella, 2018; Mirabella et al., 2022). Aside from methodological issues, appraisal theories of emotions (Moors and Fischer, 2019; Scherer and Moors, 2019) can explain literature inconsistencies. Such theories sustain that the behavioral responses elicited by valenced stimuli are not automatic or fixed but vary according to the relevance of the stimulus in a given context. For instance, the sight of a tarantula does not always cause an immediate feeling of fear and an avoidance reaction. Quite the opposite, if an entomologist is looking for a tarantula, its sight will elicit a positive emotion and an approach reaction. Recent empirical evidence provides strong support for appraisal theories of emotions. In a series of studies, Mirabella and colleagues showed that emotional facial expressions affect motor control only when task-relevant, i.e., when participants needed to pay attention to the stimuli’ valence to give a correct response (Mirabella, 2018; Mancini et al., 2020, 2022; Mirabella et al., 2022). In all studies, the experimental design consisted of giving two different versions of Go/No-go tasks to healthy participants in a counterbalanced fashion. In one version, emotions were task-relevant (Emotional Discrimination task). In the other version, emotions were task-irrelevant, i.e., even though the same pictures were shown to participants, they needed to pay attention to the actors/actress’ gender and not to the emotional expressions to respond correctly. Mirabella (2018); Mancini et al. (2020) required participants to perform a reaching movement in response to pictures of emotional facial expressions and to refrain from moving in response to neutral facial expressions in the Emotional Discrimination task. Differently, in the Gender Discrimination task, emotions were task-irrelevant, and participants had to move according to the faces’ gender, disregarding their emotional valence. Results showed that only when task-relevant threatening expressions [fear in Mirabella (2018), fear and anger in Mancini et al. (2020)] impaired motor control by increasing the reaction times (RTs) and the percentage of omission errors (i.e., instances in which participants did not move although they had to) with respect to happy faces. By contrast, the difference between happy and threatening faces disappeared when task-irrelevant. Mancini et al. (2022) found evidence that also inhibitory control is impacted by facial emotions, provided that they are task-relevant. In this study, in the Emotional Discrimination task, emotional facial expressions (fearful and happy) were shown in No-go trials. At the same time, participants had to perform reaching movements at the presentation of neutral faces. The authors showed that the percentage of commission errors, i.e., instances in which participants moved although they had not, was higher for happy than fearful faces. However, no differences between happy, fearful, and neutral faces were observed in the Gender Discrimination task. Finally, Mirabella et al. (2022) showed that whole-body movements (forward gait initiation, GI) share the same features as reaching arm movements, i.e., facial emotions altered GI parameters only when their appraisal was requested. Such finding is relevant as whole-body movements represent a more ecologically valid model for assessing the link between valenced stimuli and movement direction (Koch et al., 2009). In fact, moving the whole-body toward or away from an emotional stimulus decreases or increases the physical distance between the stimulus and the self. Importantly, in all the above-cited studies, the effects could be ascribed only to stimuli valence as other key confounding factors, such as arousal and stimulus complexity, were always carefully controlled. In sum, the impact of task relevance was shown on different aspects of motor control [motor planning (Mirabella, 2018; Mancini et al., 2020), and inhibitory control (Mancini et al., 2022)], on different effectors [arm movements (Mirabella, 2018; Mancini et al., 2020, 2022), and forward gait initiation (Mirabella et al., 2022)], and on different emotional stimuli (angry, happy, and fearful faces). These findings suggest that threatening stimuli capture and hold attentional resources more strongly than happy expressions when relevant to task performance.

Even though faces have a crucial role in nonverbal social communication allowing one to recognize others’ emotions and trustworthiness (Jack and Schyns, 2015; Crivelli and Fridlund, 2018), they are not the only socially relevant emotional stimuli. In the last decade, it has become progressively clearer that whole-body expressions are as important as faces for understanding others’ emotional states and adapting our behavior (de Gelder, 2009; de Gelder et al., 2010). However, it has also been suggested that emotional information carried by faces and bodies differs. First, bodily expressions allow recognizing emotions of individuals when others are viewed from a long distance or in a situation in which the vision of the face is occluded (de Gelder, 2009). Second, emotional body expressions are powerful conveyers of action intentions, as to perform movements, the body should be oriented in the appropriate direction. For instance, an aggressive direct-facing posture is a stronger immediate danger signal than an angry facial expression. Similarly, fearful body postures could provide information on the individual’s emotional state, the direction from which the threat originates, and how the individual copes with it. In sum, facial expressions seem to bear emotional information more related to persons’ mental states, while whole-body expressions convey emotional information more about persons’ potential actions and reactions (Calbi et al., 2017, 2021). Finally, it has also been shown that the perception of emotional facial expressions is biased by whole-body emotional expressions and vice versa (Meeren et al., 2005; Rajhans et al., 2016). Given the theoretical importance of the effect of task relevance, it is crucial to assess whether the evidence obtained with facial expressions can be generalized to emotional body postures.

**Figure 1:**
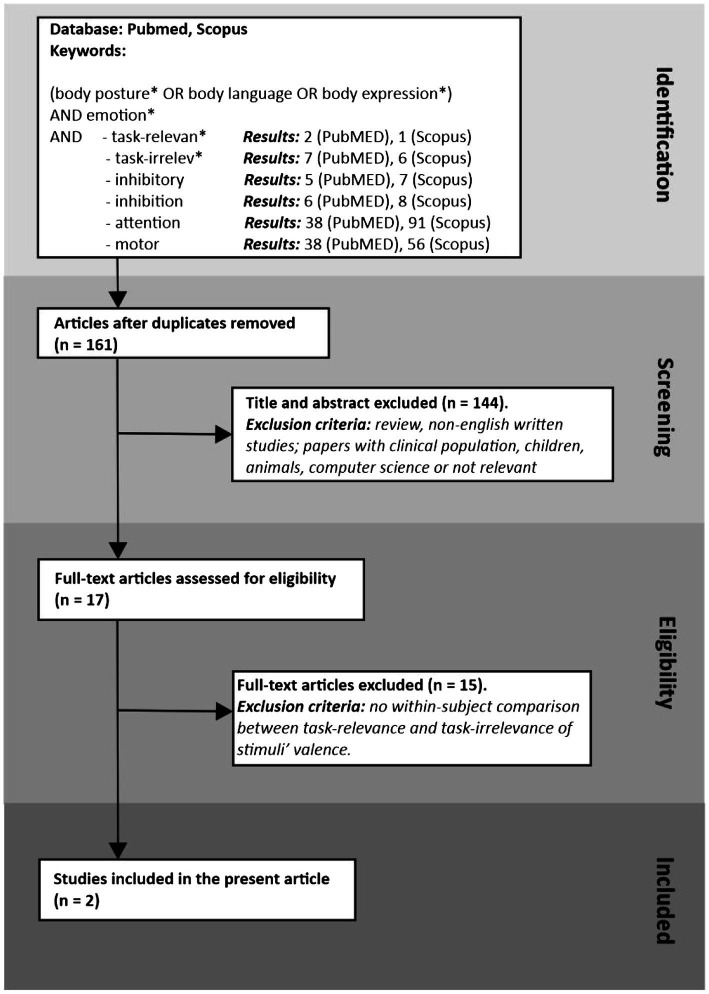
Flow charts of two systematic review procedures using PubMed (pubmed.ncbi.nlm.nih.gov/) on March 24, 2022, and Scopus (www.scopus.com/) on March 25, 2022. Separately on the two databases, we performed six different searches using keywords to be found in the titles or abstracts. All searches differed only for the last keyword. After removing duplicates, we analyzed the titles and abstracts, excluding studies on populations not of interest, reviews, non-English written, or papers not relevant to the current topic. The remaining articles were full-examined, and we excluded articles that did not include a within-participants comparison between two conditions of task relevance. The final result of our search included two studies.

To search for articles dealing with the effect of task relevance on emotional body postures, we performed a literature search using PubMed and Scopus to identify studies exploiting a within-subject design in which participants were tested when the stimuli’ valence was relevant for the task and versus when it was not. As Figure 1 shows, we found just two studies that satisfied our stringent criteria.

Oldrati et al. (2019) investigated whether the processing of emotional information conveyed by facial and body expressions is automatic, i.e., pre-attentive or not. To this end, they performed two experiments. In the first one, they gave an Eriksen Flanker Task (Eriksen and Eriksen, 1974) to 24 participants that were asked to discriminate either the emotion (Emotion task) or the gender (Gender task) of the body/face shown in the middle of a three-stimulus array while ignoring the lateral, i.e., the flanker, images. The key finding was that, independently of the type of stimuli and the task, when the valence of the central and lateral images was congruent, participants were more efficient, i.e., faster and more accurate, in indicating the emotion than when the valence was incongruent. Differently, the congruency/incongruency of the gender never impacted the performance. These results were interpreted as the emotional value of the stimuli could not be filtered out even when it was task-irrelevant. Interestingly, however, Oldrati et al. (2019) obtained different results in a second experiment in which participants were required to match the emotion or the gender of the central and lateral stimuli. They found that, during the Gender Task, participants were less efficient in giving a gender judgment when the valence of the middle images was incongruent with that of the flankers, provided that pictures represented body postures. Thus, in this case, the emotional features impaired the same-different judgments only for bodies but not facial expressions. By contrast, the study of Gandolfo and Downing (2020) showed that judgments of emotions expressed by body pictures are not influenced by the actors’ gender and vice versa. Therefore, they concluded that gender and emotions expressed by the whole-body are processed independently, i.e., the stimuli’ emotional dimension can be disregarded when task-irrelevant.

Direct evidence about the effect of task relevance on motor responses elicited by body postures is very scarce and provides contrasting results. Differently, recent research on facial expressions (Mirabella, 2018; Mancini et al., 2020, 2022; Mirabella et al., 2022) showed that all aspects of motor control, i.e., planning, execution, and inhibition, are affected by emotions only when participants have to pay attention to such stimuli features. However, as both faces and bodies are potent conveyors of emotional signals, it is crucial to assess whether this phenomenon can be generalized to emotional body postures or, conversely, it is restricted to emotional facial expressions. To shed light on this issue, we employed the experimental paradigm of Mancini et al. (2022) to investigate for the first time whether fearful and happy bodily postures modulate inhibitory control of arm movements. Thus, we gave participants two versions of a Go/No-go task. In the Emotional Discrimination task, they were instructed to refrain from moving at the presentation of images of fearful and happy bodily expressions and moving at the presentation of neutral postures. In contrast, in the control version (Color Discrimination task), participants were instructed to respond according to the color of the actor/actress’ t-shirt (i.e., beige and lilac), disregarding the body’s emotional expressions. In previous studies (Mirabella, 2018; Mancini et al., 2020; Mirabella et al., 2022), we found that when facial emotions are task-relevant and emotions provide the Go-signal, fearful and angry faces increased the RTs and the rate of omission errors, i.e., instances in which participants did not move toward the peripheral target even though they had to. We interpret these findings, suggesting that threatening expressions capture and hold attention more than happy faces, possibly to detect the source of potential threats. On this ground, we hypothesize that if facial emotions are task-relevant and emotions provide the No-go-signal, threatening faces should allow a better inhibitory control than happy expressions. This is what Mancini et al. (2022) found. Therefore, in line with the results of Mancini et al. (2022), in the current study, we expected that when task-relevant, the presentation of fearful body postures captures and holds participants’ attention allowing for better inhibitory control than happy body postures. Instead, we did not expect to find any difference in inhibitory proficiency in the Color Discrimination task.

## Materials and methods

In the present section, we report information about sample size, participants’ inclusion and exclusion criteria, data rejection and the type of statistical analysis to be applied. All these parameters were established in advance.

### Participants

The sample size was estimated with GLIMMPSE 2.0 (Kreidler et al., 2013), using data from a similar study with facial expressions (Mancini et al., 2022) as there was no data available in the literature on body postures. The output established that a minimum sample of 35 participants was required, considering a power of 0.95, a Type I Error Rate of 0.05, a Means Scale Factor of 0.5, and a Variability Scale Factor of 2. Analysis was performed on the interaction effect between Task (2 levels) and Emotion (2 levels) and with a Univariate Approach to Repeated Measures with Greenhouse–Geisser Correction. Therefore, we recruited 38 healthy volunteers. All the participants were right-handed, as assessed by the Italian version of the Edinburgh handedness inventory (Oldfield, 1971), with a normal or corrected-to-normal visual acuity, without a history of psychiatric and neurological disorders, and were naïve about the purpose of the study. Two participants were discarded due to outlier values of the rates of omission errors (Mean= *M;* *M*_participant1_ = 19.3%, *M*_participant2_ = 31%; *M* ± Standard Deviation (*SD*) of the sample: = 5.6 ± 5.6%) in the Emotional Discrimination task during Go-trials. Such an elevated rate of omission errors reveals a strong tendency of these participants to postpone their response to make inhibition on No-go trials easier, despite verbal instruction. Thus, the final sample was constituted of 36 healthy participants (18 females, *M* ± *SD* age = 25.9 ± 4.4 years, range = 18–35 years). All participants provided written informed consent to participate in the study, which was approved by the local ethical committee “ASST Spedali Civili” of Brescia, Italy (protocol number 4452) and was conducted in accordance with the Declaration of Helsinki 2013.

### Stimuli

**Figure 2:**
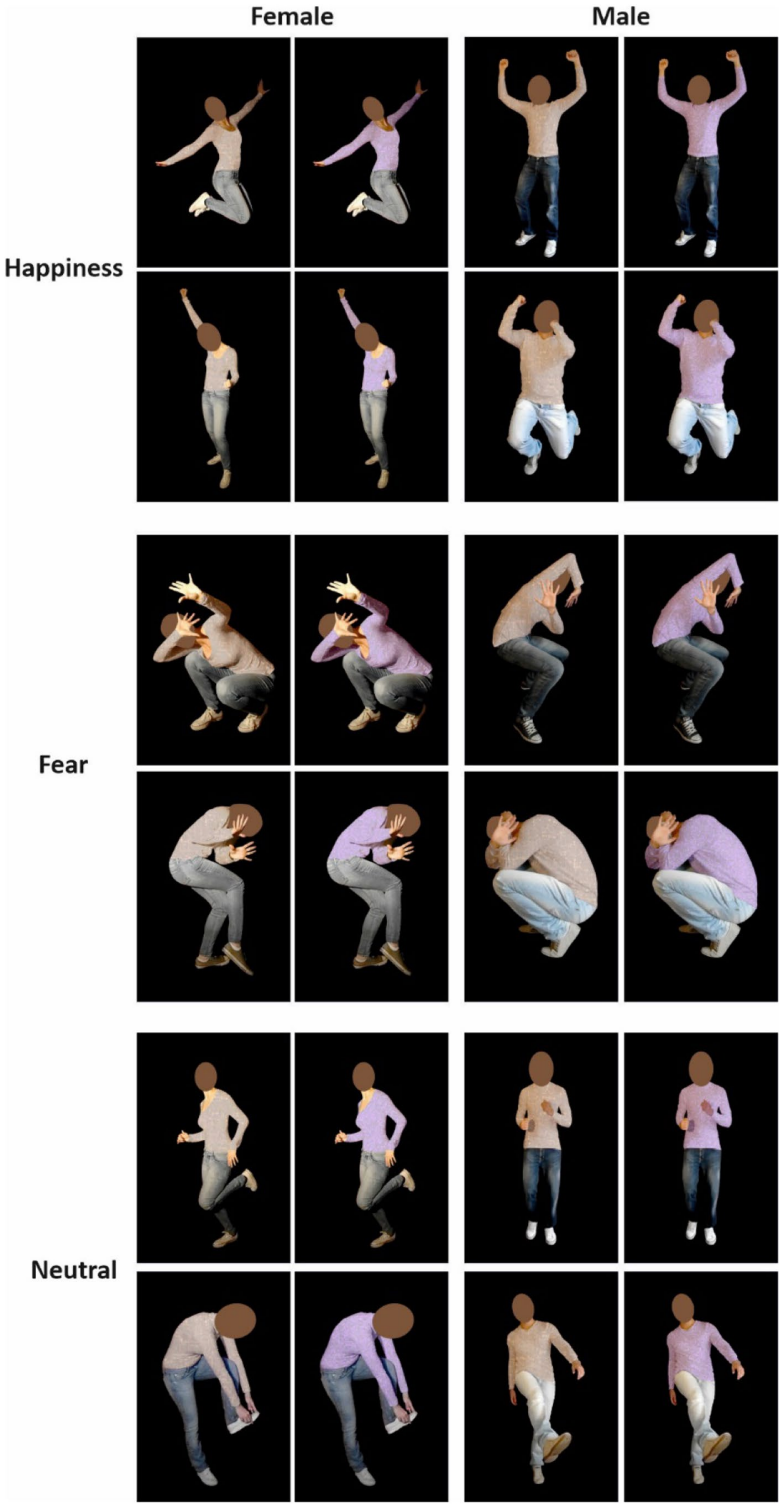
Twelve colored pictures were taken from Mazzoni et al. (2022) database. Photos displayed the bodies of two actors and two actresses with blurred faces on a black background. They enacted emotional postures (i.e., fear and happiness) or neutral. Neutral stimuli were dynamic body postures in which actors/actresses were performing actions without emotional connotations, such as running, kicking, or wearing a sock. Thus, our final set of stimuli was composed of 24 images.

Stimuli were taken from Mazzoni et al. (2022) and consisted of 12 pictures displaying the bodies of four actors (two females) enacting fearful, happy, and neutral postures with blurred faces on a black background. In all pictures, individuals performed meaningful actions so that both emotional and neutral images illustrated biological movements. Neutral body actions included the pantomime of wearing a sock, kicking a ball, and jogging. In order to have a set of stimuli enabling us to run a control task (Color Discrimination task, see the Procedure), we colored stimuli’s t-shirts using Photoshop (CC 2019) in beige and lilac. We avoided red and green because some studies claimed association with stop and go signals (Blizzard et al., 2016; Kubo et al., 2021) or with altered emotion perception (Gil and Le Bigot, 2015). Therefore, our final stimuli set was composed of 24 images (Figure 2). At the end of the experimental session, participants were asked to rate the valence and arousal of each stimulus using a Visual Analogue Scale (Numerical rating scale). Valence ranged from 0 = negative to 100 = positive (50 = neutral). Arousal ranged from 0 = not at all arousing to 100 = very much arousing. As the Shapiro–Wilk tests showed that not all images’ valence ratings were normally distributed (Happiness: *W* = 0.97; *p* = 0.39; Fear: *W* = 0.82; *p* < 0.0001; Neutral: *W* = 0.93; *p* = 0.03), we used a non-parametric Friedman rank-sum test to compare the stimuli’ valence with Emotion as a factor (Fear, Happiness, and Neutral). We found a significant main effect [*χ*^2^(2) = 72, *p* < 0.0001]. *Post hoc* comparison with Bonferroni correction revealed that fearful body postures had significantly lower valence than neutral (Fear, *M* ± *SD* = 9.1 ± 8.8; Neutral, *M* ± *SD* = 53.8 ± 6.2) and happy ones (*M* ± *SD* = 83.6 ± 9.5). In addition, happy body postures had a significantly higher valence than neutral ones (all *p_s_* < 0.001). Given that also not all images’ arousal ratings were normally distributed, as assessed by Shapiro–Wilk tests (Happiness: *W* = 0.95; *p* = 0.15; Fear: *W* = 0.88; *p* = 0.001; Neutral: *W* = 0.94; *p* = 0.07), we performed the same analysis on stimuli’ arousal. We found a significant main effect of Emotion again [*χ*^2^(2) = 47.72, *p* < 0.0001]. *Post hoc* comparison with Bonferroni correction revealed the arousal rating for all body postures categories was different (Fear, *M* ± *SD* = 80.4 ± 13; Happiness, *M* ± *SD* = 75.5 ± 9.9; Neutral, *M* ± *SD* = 39.4 ± 19.3; all *p_s_* < 0.05).

### Experimental apparatus and procedure

Participants were comfortably seated in a quiet and dimly illuminated room. Visual stimuli were projected onto a 28′′ screen with a 1,920 × 1,080 pixel resolution, positioned about 60 cm away from the participant. Images all had the same dimension (400 × 600 pixels or 12.29 × 17.89 degrees of visual angles). The experimental paradigm consisted of two versions of a Go/No-go task, i.e., the Emotional and the Color Discrimination tasks, following the logic of Mirabella (2018) and Mancini et al. (2022). Both tasks were run in a single experimental session, and the presentation order was counterbalanced across participants. Before each experimental session, a short training session was included to familiarize participants with the tasks. To avoid habituation, all the training trials included body posture images that were not used in the experimental task. The experimenter was present in the room only during the training session to ensure that tasks were clearly understood. However, participants’ performance was continuously controlled *via* a closed-loop video camera.

Stimuli presentation and behavioral responses were controlled using E-prime software (version 2.10; Psychology Software Tools, Inc.) running on a PC.

### Emotional discrimination Go/No-go task

**Figure 3:**
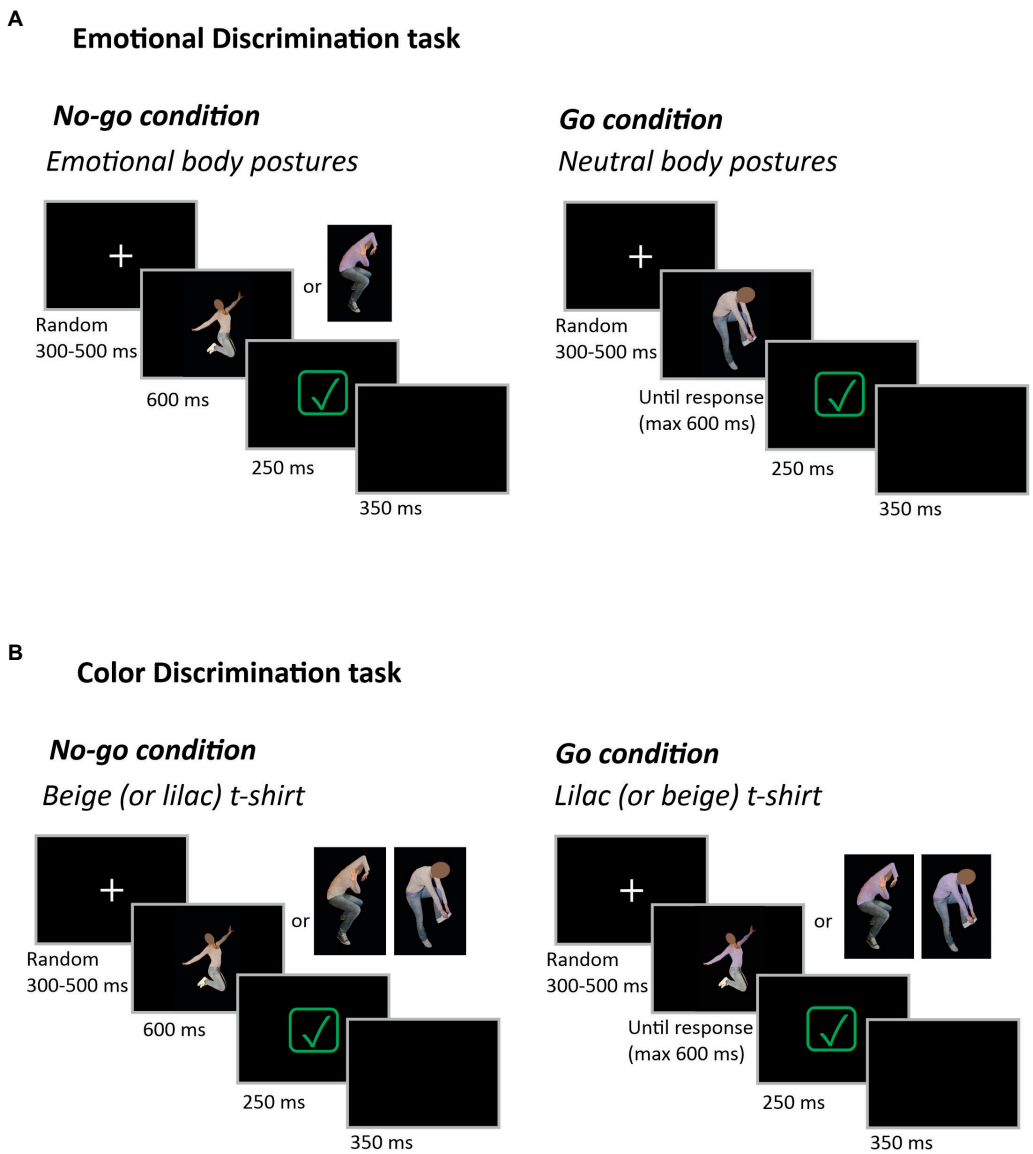
Experimental Design. Emotional Discrimination task **(A).** Each trial started with a fixation cross at the center of the screen. After a random delay of 300–500 ms, the picture of a body posture, either fearful, happy, or neutral, was shown. Participants were instructed to respond as quickly and accurately as possible to neutral stimuli (Go-signal, 67%) by pressing the mouse’s left button with their right index finger. Differently, they had to refrain from responding when happy and fearful postures were presented (No-go signal, 33%). Visual feedback, i.e., a green checkmark, signaled correct trials. The inter-trial interval was 350 ms. Color Discrimination task **(B)**. The sequence of events was the same as in **(A)**, except that participants were instructed to respond or not according to the color of the actor/actress’ t-shirt. To avoid a color bias, one-half of the participants had to respond to the appearance of a beige t-shirt, withholding it when a lilac t-shirt was shown, and vice versa for the other half of participants. We used stimuli from Mazzoni et al. (2022), which can be freely published. See the online version of the present article for colored figures.

All trials started with a white fixation cross on a black uniform background (Figure 3A). Participants had to fixate it for a random interval of 300–500 ms, then one image showing a body posture (fearful, happy, or neutral) was shown. Participants were instructed to respond as quickly and accurately as possible to the presentation of neutral body postures (Go-trial, 67% of the total trials) by pressing the left mouse button with their right index. Instead, they had to withhold their response when an emotional body posture was shown (No-go trial, 33% of the total trials). Correct trials were signaled by a green checkmark on black background, lasting 250 ms. The inter-trial interval lasted 350 ms. Participants had a time limit of 600 ms to respond to Go-signals (upper-reaction time, RT). Responses exceeding the upper-RT were considered errors. However, we gave participants an extra time of an additional 100 ms to press the mouse button with the purpose of not cutting the RTs’ right tail distribution (overtime-trials; see Mirabella et al., 2006; Mirabella, 2018; Mancini et al., 2022). The RTs of overtime-trials were included in the analyses, and such trials accounted for 12.11 ± 7.49% of the total Go trials.

Each neutral body posture (*n* = 8) was repeated 48 times for a total of 384 Go trials, while fearful and happy body postures (*n* = 16) were repeated 12 times for a total of 192 No-go trials. All experimental conditions were randomized, and the task was divided into two blocks of 288 trials. In each block, the proportion between Go and No-go trials was maintained (2:1), and emotional body postures and t-shirts’ colors were balanced. Participants could rest at the end of each block of trials when requested.

### Color discrimination Go/No-go task

The task structure was identical to the Emotional Discrimination Go/No-go task, except for the fact that participants had to respond or refrain from their responses according to the color of the actor/actress’ t-shirt (i.e., beige and lilac, Figure 3B). To avoid a color bias, one-half of the participants had to respond to the appearance of beige t-shirts and withhold the response when lilac t-shirts were shown. Vice versa for the other half of the participants. The RTs of overtime-trials were included in the analyses, and they accounted for 2.1 ± 2.5% of the total Go-trials. Participants performed 576 trials in two blocks of 288 trials. The frequency of the Go-trial was 67%, thus each image (*n* = 12) was repeated 32 times for a total of 384 Go-trials. In No-go trials, each image (*n* = 12) was repeated 16 times for a total of 192 trials. In each block, the proportion between Go and No-go trials was maintained (2:1), and emotional body postures and t-shirts’ colors were balanced. All experimental conditions were randomized.

### Data analysis

As a behavioral index of response inhibition proficiency, we used the percentage of commission errors in No-go trials. Separately for each participant and experimental condition, we computed the commission error rates as the ratio between the number of commission errors in a condition and the total number of trials in the same condition multiplied by 100.

Shapiro–Wilk test assessed normality. We found that the two variables of the Emotional Discrimination task were normally distributed (Shapiro–Wilk test: (i) Happiness: *W* = 0.98; *p* = 0.75; (ii) Fear: *W* = 0.98; *p* = 0.62); while the three variables of the Color Discrimination task were not normally distributed (Shapiro–Wilk test (i) Happiness: *W* = 0.72; *p* < 0.0001; (ii) Fear: *W* = 0.78; *p* < 0.0001; (iii) Neutral: *W* = 0.82; *p* < 0.0001). Thus, as parametric tests are robust to violation of normality (Blanca et al., 2017) we used a parametric test when comparing the effect of Fear and Happiness in the Emotional vs. the Color Discrimination task. By contrast, when comparing the effect of Fear, Happiness, and Neutral in the Color Discrimination task, as all the variables were not normally distributed because participants made too few errors, we controlled the floor effect using the Brown–Forsythe F star test available in the DACF package for R (Liu and Wang, 2021). A 3-way ANOVA with a mixed design [between-participant factor: Delta Arousal (2 levels: High Arousal, Low Arousal), within-participant factors: Emotion (2 levels: Happiness, Fear) and Task (2 levels: Emotional Discrimination task, Color Discrimination task)] was performed to analyze the average rate of commission errors across experimental conditions. Bonferroni corrections were applied to all *post hoc* tests. We included the Delta Arousal (see below) as between factor because participants’ ratings of arousal differed for the two emotions. We compared arousal ratings for happy and fearful body postures using the Revised Standardized Difference Test (RSDT; Crawford and Garthwaite, 2005). The RSDT analysis evaluates the individual’s differences by assessing whether the standardized difference between individuals’ ratings differed significantly from the average difference of the other n-1 judgments considered like a control group. These *z* values are then converted into percentiles, creating an index that we call Delta Arousal, which represents the rarity of the individual’s difference, expressed as a proportion of the population with a greater discrepancy. To create two equally sized subgroups, participants with a Delta Arousal comprised between the 30th to 70th percentile were included in the ‘Low arousal’ group, while the others were included in the ‘High arousal’ group. To compare commission error rates across the three conditions in the Color Discrimination task, we used the Brown–Forsythe F star test, which allows for comparing variables that are adjusted for ceiling and floor effects through the estimation of means and variances (Liu and Wang, 2021). Finally, to compare the RTs of go-trials in the Emotional Discrimination task vs. the Color Discrimination task, we used a paired *t*-test. Instead, we used a Wilcoxon signed-rank test to compare the omission errors in go-trials.

The effect sizes were reported as partial eta-squared and Cohen’s *d*. Bayes Factors (BF_10_; Jarosz and Wiley, 2014) were computed with an r-scale of 0.707 to quantify the null hypothesis’ strength (R package BayesFactor; Morey and Rouder, 2018). Values of BF_10_ > 3 and > 10 indicate moderate and strong support for the alternative hypothesis, respectively. Values of BF_10_ < 0.1 and < 0.33 indicate strong and substantial support for the null hypothesis; and values 0.33 < BF_10_ < 3 are inconsistent for any hypothesis.

All statistical analyses were made using R, version 4.0.0 (R Core Team, 2020).

## Results

### Analyses of commission errors

**Figure 4:**
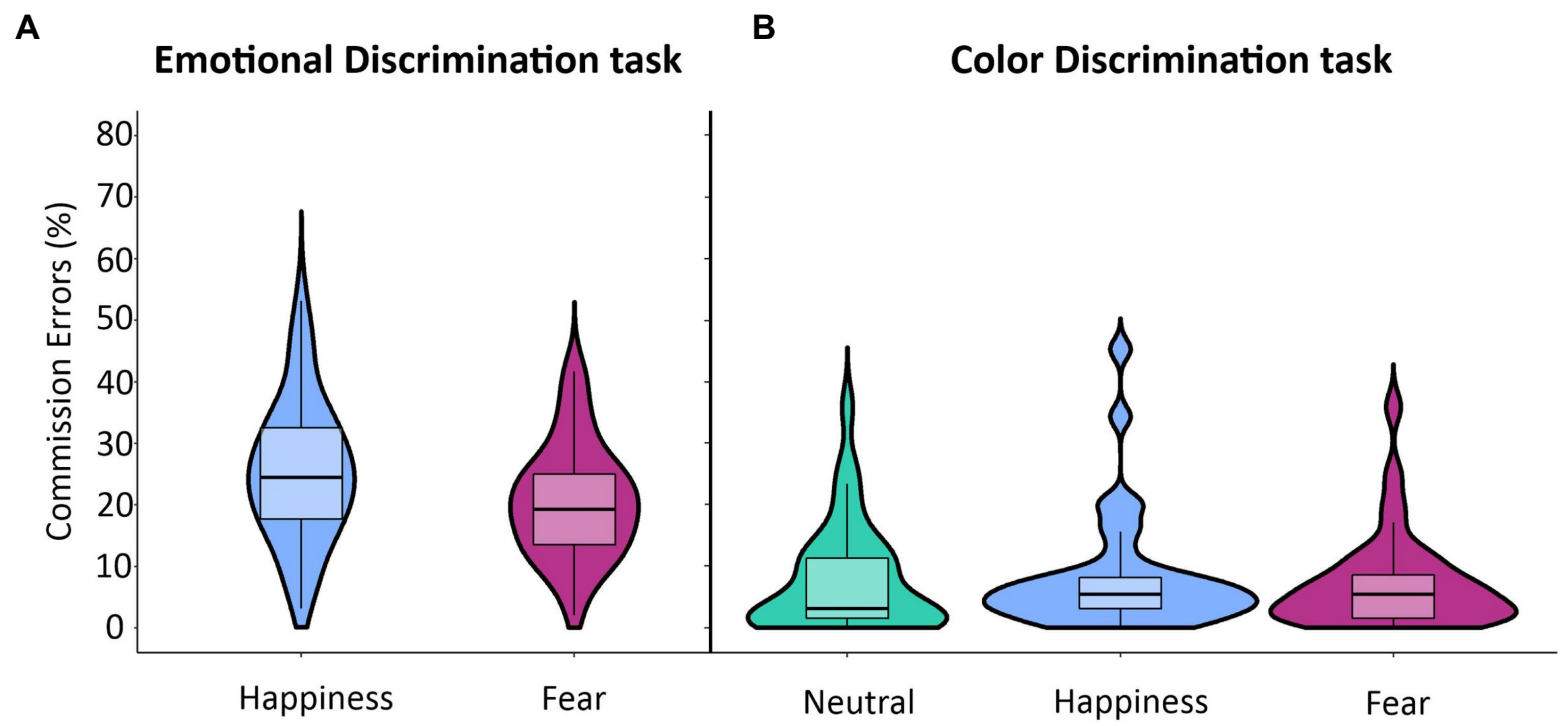
Effects of emotional body postures on average commission errors in the Emotional and Color Discrimination tasks. In the Emotional Discrimination task **(A)**, the rate of commission errors was significantly higher for happy than for fearful body postures (see Table 1 for statistics). Differently, in the Color Discrimination task **(B)**, there were no differences in the rates of commission errors between any of the three types of body postures. The violin plots depict kernel probability density, i.e., the width of the areas represents the relative frequency of the data. Box plots are shown inside the violin plots. The lower box’s boundary indicates the first quartile, the median is marked with a black line.

We assessed the effect of task relevance of emotional body postures on motor inhibition by comparing the commission error rates across the Emotional Discrimination task and the Color Discrimination task using a 3-way ANOVA with a mixed design (see Table 1; Figure 4). We found a significant main effect of Task (*F*(1,34) = 82.78; *p* < 0.001), which was due to a higher commission error rates in No-go trials in the Emotional (*M* = 22.9; *CI* = 19.6–26.3) than in the Color Discrimination task (*M* = 7.6; *CI*  = 4.8–10.4). We also had a significant main effect of Emotion (*F*(1,34) = 15.98; *p* < 0.001) due to the fact that the commission error rate was higher for happy (*M* = 16.8; *CI*  = 13.9–19.6) than to fearful body postures (*M* = 13.8; *CI*  = 11.4–16.3). These effects were qualified by the significant interaction between the factors Task and Emotion (*F*(1,34) = 8.20; *p* < 0.01). *Post hoc* comparisons showed that the commission error rates were significantly higher for happy (*M* = 25.4; *CI*  = 21.5–29.3) than for fearful (*M* = 20.5; *CI*  = 17.3–23.7) body postures just in the Emotional-discrimination task. No difference occurred in the Color Discrimination task between happy (*M* = 8.2; *CI*  = 5.0–11.3) and fearful (*M* = 7.1; *CI*  = 4.6–9.7) body postures. Notably, we did not find any significant effect of Delta Arousal.

**Table 1:** Results of the statistical analyses on commission error rates.

Effect	Factors	Statistics	*p* value	*ES*	BF_10_
Main	Delta arousal	F(1,34) = 1.05	0.31	*η*p^2^ = 0.03	0.51
Main	Task	F(1,34) = 82.78	**<0.001**	*η*p^2^ = 0.71	>100
Main	Emotion	F(1,34) = 15.98	**<0.001**	*η*p^2^ = 0.32	3.27
Interaction	Delta arousal ^*^task	F(1,34) = 0.40	0.53	*η*p^2^ = 0.01	0.36
Interaction	Delta arousal ^*^emotion	F(1,34) = 1.04	0.31	*η*p^2^ = 0.03	0.28
Interaction	Task ^*^emotion	F(1,34) = 8.20	**0.007**	*η*p^2^ = 0.19	0.87
Interaction	Delta arousal ^*^task ^*^emotion	F(1,34) = 0.12	0.73	*η*p^2^ = 0.00	0.22
*Post hoc* comparison	Task: Emotional vs. Color	*t*(34) = −9.10	**<0.0001**	*d* = −1.52	>100
*Post hoc* comparison	Emotion: Fear vs. Happiness	*t*(34) = −4.00	**<0.001**	*d* = −0.67	>100
*Post hoc* comparison	Interaction task ^*^emotion				
	EMO Fear–EMO Happiness	*t*(34) = −4.02	**0.001**	*d* = −0.67	99.3
	COL Fear–COL Happiness	*t*(34) = −1.45	0.63	*d* = −0.24	0.47
	COL Fear–EMO Fear	*t*(34) = −8.96	**<0.001**	*d* = −1.50	>100
	COL Happiness–EMO Happiness	*t*(34) = −8.29	**<0.001**	*d* = −1.38	>100

*Delta Arousal* = index of the arousal difference between fearful and happy body stimuli (see text for more details); *ES* = effect size, partial eta squared (*η*p^2^) for the ANOVAs and Cohen’s *d* for the *post hoc* tests; *BF_10_* = Bayes Factors report the ratio of likelihood of the alternative hypothesis to the likelihood of the null hypothesis; *p*-values were reported in bold when < 0.05; alpha level in *post-hoc* (i.e., pairwise) comparisons were adjusted according to Bonferroni correction.

It should be stressed that even though the inferential statistic indicates a significant effect (*p* = 0.007), the BF_10_ of the interaction Task*Emotion is 0.87, i.e., it means inconsistency for any hypothesis. However, the crucial tests to interpret the effect of emotional body postures on inhibitory control are the *post hoc* tests, where the percentage of commission errors to fearful and happy body postures in the Emotional and the Color Discrimination task are compared. Both cases are fully congruent. In the Emotional Discrimination Task, both the inferential (*p* < 0.001) and the Bayesian statistics (BF_10_ = 99.3) strongly sustain the alternative hypothesis. Conversely, in the case of the Color Discrimination Task, they both support the null hypothesis (see Table 1).

**Figure 5:**
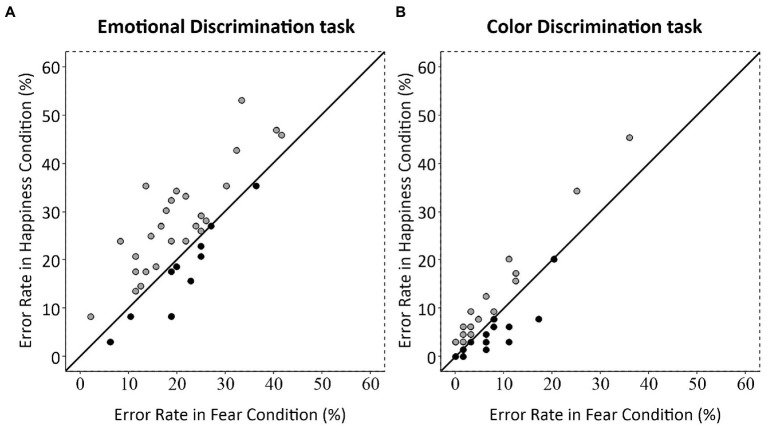
Scatterplot of commission errors for happy and fearful postures for each participant in the Emotional and Color Discrimination tasks. Each dot represents one participant. **(A)** In the Emotional Discrimination task, most of the participants (26/36 or 72%) are located above the first quadrant angle’s bisector, showing a bias towards a higher rate of commission errors for happy than for fearful postures. **(B)** In the Color Discrimination task, participants are equally distributed above and below the bisector line, indicating a similar commission error rate for the two bodily expressions in the Color Discrimination task.

To check whether the rates of commission errors for fearful and happy body postures were different from neutral body postures, we ran a Brown–Forsythe F star test [Emotion (3 levels: Happiness, Fear, Neutral)]. It did not show any significant effect (*F* = 0.22, *p* = 0.64). Finally, we analyzed the effect of emotional body postures at the individual level by correlating the average rates of commission errors for happy and fearful postures obtained for each participant in the two tasks. In both the Emotional Discrimination task [Spearman’s *ῥ*(34) = 0.70; *p* < 0.0001; Figure 5A] and Color Discrimination task [Spearman’s ῥ(34) = 0.75; *p* < 0.0001; Figure 5B] the correlations were significant, meaning that single individuals were either accurate or not on both emotional postures. Nevertheless, in the Emotional Discrimination task, 26 participants out of 36 (72.2%) had a higher commission error rate for happy than fearful body postures, nine participants (25%) showed the opposite pattern, and one participant (2.8%) had the same commission error rate for the two emotional body postures. Chi-square goodness of fit test, where the participant showing the same rate of commission errors was excluded, indicated that participants with a higher commission error rate for happy postures were significantly more than those with a higher commission error rate for fearful postures [*χ*^2^ (1) = 8.26, *p* < 0.01]. In the Color Discrimination task, 19 participants out of 36 (52.8%) had a higher commission error rate for happy than fearful body postures, 11 (30.5%) had the opposite pattern, and six (16.7%) showed the same commission error rate for happy and fearful body postures. Excluding participants with the same commission error rate, the frequency of participants with different commission error rates was not different [χ^2^(1) = 2.13, *p* = 0.14].

### Analyses of RTs and omission error rates of go-trials

To provide a full overview of the data, we computed the RTs and omission error rates for the Go-trials in the Emotional Discrimination task (RTs: *M* ± SD = 424.3 ± 26.8 ms; omission errors: *M* ± SD = 4.5% ± 2.9%) and for the Color Discrimination task (RTs: *M* ± SD = 359.5 ± 23 ms; omission errors: *M* ± SD = 0.7% ± 1%). Notably, while in the Emotional Discrimination task, the Go-signal was always a neutral body posture, in the Color Discrimination task, it could be either a neutral, a happy, or a fearful posture. A paired *t*-test showed that RTs in the Emotional Discrimination tasks were significantly slower than those in the Color Discrimination task [*t*(35) = 13.81; *p* < 0.0001]. In addition, participants made more omission errors in the Emotional than in the Color Discrimination task (Wilcoxon signed rank test, *W* = 661; *p* < 0.0001). This evidence indicates that the Color Discrimination task was easier than the Emotional Discrimination task. We will not discuss these data further.

## Discussion

In this study, for the first time, we compared the impact of the same emotional body postures on inhibitory control in two different experimental conditions, i.e., when they were relevant to the responses required to the participants versus when they were not. In line with our predictions, results showed that when the instruction was to refrain from moving at the presentation of one of the two emotional expressions, happy body postures impaired inhibitory control with respect to fearful body postures. By contrast, when the instruction was to inhibit the movement according to the color of the actor/actress’ t-shirt, the valence of body expressions did not yield any effect. This evidence indicates that, as for facial expressions (Mirabella, 2018; Mancini et al., 2020, 2022; Mirabella et al., 2022), the appraisal of the body postures’ valence is not automatic. Instead, it depends on the relevance of the stimuli’ emotional content for participants’ goals, supporting the claim of appraisal theories of emotions (Moors and Fischer, 2019; Scherer and Moors, 2019).

We trust our results for several reasons. First, we adopted a within-design, and our sample was larger than in previous similar studies (Oldrati et al., 2019; Gandolfo and Downing, 2020). Second, visual features cannot explain the results, as the same pictures were shown in the Emotional and Color Discrimination tasks. In addition, differences in visual elements between happy and fearful body images could be ascribed only to the enacted emotional expressions, not to other characteristics, as could be the case of the International Affective Picture System (Lang, 2005). In such a database, positively and negatively valenced stimuli differ also in terms of visual features (e.g., the subjects could be humans, animals, parts of the body, or objects; sometimes they are in the foreground, some other times in the background). Third, our analyses allow us to exclude that images’ arousal could impact our findings. Fourth, Bayesian factors strongly support the difference between commission error rates in response to fearful and happy body postures in the Emotional Discrimination task (BF_10_ > 10) and provide reasonable support to the absence of such difference in the Color Discrimination task. Note that Bayes Factors represent continuous evidence. Thus, a BF_10_ of 0.47 indicates only slightly less moderate evidence than a Bayes Factor of 0.33.

### Could attention filter out task-irrelevant emotions?

The idea that emotional responses, especially those linked to our biological fitness (e.g., those occurring when in danger or when hungry or thirsty), have been sculpted in ‘survival circuits’ throughout evolution (LeDoux, 1996, 2012) profoundly influenced the interpretation of behavioral reactions to valenced stimuli. In line with this hypothesis, it has been suggested that our attentional system has been shaped for rapid and automatic detection of emotional stimuli, especially threatening ones, and for eliciting adaptive behaviors independently of the individual’s willingness (Bradley et al., 2001; Vuilleumier, 2005; Lang and Bradley, 2010). However, the empirical evidence about how emotional expressions influence attentional deployment, action preparation, or response inhibition is highly inconsistent in that the response to emotional stimuli greatly varies. Scrutinizing again the papers available in the literature found with the systematic review illustrated in Figure 1, we identify seven studies in which participants had to provide a manual response to the presentation of emotional body images. Two studies did not report the RTs to different categories of emotional stimuli (e.g., fearful or happy body expressions Oldrati et al., 2019; Gandolfo and Downing, 2020). The five remaining works provided highly contrasting results as the effect of body expressions changes continuously (see Table 2). For instance, sometimes fearful body postures induce shorter RTs, but other times fearful body postures induce longer RTs than other body expressions. In addition, the fearful bodies’ effect also depends on the presentation times.

**Table 2:** Effect on manual reaction times (RTs) to the presentation of emotional body images (see text for more details).

	Task	Emotional body postures	Effect on RTs	Relevance of emotional stimuli
de Valk et al. (2015)	Dot-probe task (participants had to touch a dot and then the emotional image)	Fearful, angry, and neutral	Angry<fearful and neutral (which did not differ)	irrelevant
Botta et al. (2021)	Forced-choice task (emotion recognition)	Fearful, happy, and neutral	Fearful<happy<neutral	relevant
Bannerman et al. (2009)	Forced-choice task (emotion recognition)	Fearful, and neutral	Fearful<neutral	relevant
			(if images presented for 500 ms)	
			Fearful = Neutral	
			(if images presented for 20 ms)	
Bannerman et al. (2010)	Cueing task (emotional bodies cued target location)	Fearful, and neutral	Emotional cues had no effect	irrelevant
			(if cue presented for 20 ms)	
			Fearful cues induces faster reactions	
			(if cue presented for 100 ms)	
Van den Stock et al. (2007)	Match to sample task (participants had to match the sample emotion among two possibilities)	Fearful, angry, happy, and sad	Sad < happy < angry < fearful	relevant

Several methodological features can explain such a high variability. First stimuli’ arousal either has not been measured or when has been assessed even though happy postures had higher arousal than fearful postures; this dimension was not considered in the analyses of motor performance (e.g., Botta et al., 2021). Thus, it could not be disentangled whether behavioral effects are due to the valence or the arousal of the emotional body postures. Nevertheless, it is known that arousal impacts motor response modulation (Lundqvist et al., 2014). Second, in some studies, emotional bodies are task-relevant (Van den Stock et al., 2007; Bannerman et al., 2009; Botta et al., 2021), in other studies, they are not (Bannerman et al., 2010; de Valk et al., 2015). Third, different cognitive processes besides action planning, e.g., working memory, can impact emotional processing when asking for explicit emotion recognition. Furthermore, explicitly labeling others’ emotional expressions is not an ecological task. In real life, sensory information about others’ emotions is used to drive actions implicitly. Instead, asking a judgment requires the conscious participation of the participant. Thus, different neural processes are likely to occur.

This observation also applies to another set of studies assessing the effect of emotional body postures on corticospinal excitability (Borgomaneri et al., 2015, 2017, 2020). In these investigations, body emotional images were shown to healthy people, asking them to categorize the emotion depicted. After showing the pictures, they delivered magnetic pulses before the participants’ responses at variable times. Borgomaneri et al. (2015, 2017, 2020) found that corticospinal excitability was suppressed already at 70–150 ms only after the onset of fearful and happy body postures but not after neutral postures. They interpreted these early modulations as a reflex of automatic emotion-related actions. However, the extent to which changes in cortical excitability of a few upper limb muscles correspond to modulations of motor behaviors is largely unclear. Second, no experiment with task-irrelevant emotional stimuli was carried on. Thus, it is not known whether changes in corticospinal excitability occur only when participants have to recognize an emotion explicitly. In sum, current research does not support the idea that threatening body postures automatically attract attention, allowing quicker responses to potentially dangerous agents.

Two studies tried assessing the role of task relevance using a within-participant design. One is in line with our results (Gandolfo and Downing, 2020), suggesting that attention can filter out task-irrelevant emotions, while the latter does not (Oldrati et al., 2019). The interpretation is difficult as both studies have limitations. The arousal dimension was not considered, and the number of trials was lower than in our tasks (fewer trials lead to a more variable estimate of the studied variables). The emotional and gender tasks of Gandolfo and Downing (2020) consisted of 128 trials each, and those of Oldrati et al. (2019) consisted of 192 trials each. By contrast, in the present paper, the Emotional and Color Discrimination tasks consisted of 384 trials each. In both previous works, participants were asked to categorize either the actor/actress’ gender or the specific emotion they enacted. In our study, participants were not asked to provide judgments but to perform a motor response according to any emotion displayed by the actors/actresses or the color of their t-shirt. Finally, the key experiments by Oldrati et al. (2019) had a smaller sample size than ours (24 vs. 36 participants). Leaving aside these facts, there is another way to interpret the similarity and differences of previous research with respect to our results. Gandolfo and Downing (2020) showed images of body postures one at a time, as we did. Differently, Oldrati et al. (2019), in each trial, presented an array of three pictures and, under these conditions, flanker images could alter the attention control system, which cannot suppress emotional task-irrelevant information. Future studies are warranted to assess this hypothesis.

All in all, the existing evidence does not hint at how emotional stimuli modulate attention. In striking contrast, Mirabella and colleagues’ Go/No-go paradigm (Mirabella, 2018; Mancini et al., 2020, 2022; Mirabella et al., 2022) yields highly reproducible results indicating that attention can filter out irrelevant emotions. The present study showed that the whole body’s emotional postures provide the same results obtained with facial expressions, indicating that such a phenomenon can be generalized across different types of emotional stimuli.

### Happy body postures impair inhibitory control when task-relevant

Results fully confirm our initial hypothesis, i.e., when task-relevant, the presentation of happy body postures impaired inhibitory control more than fearful postures. This evidence is perfectly in line with the findings of Mancini et al. (2022), indicating that the observed phenomenon goes beyond the type of stimulus employed. In keeping with the interpretation of our previous results (Mirabella, 2018; Mancini et al., 2020, 2022), we suggest that when fearful expressions are task-relevant, they draw attentional resources more efficiently than happy facial or bodily emotional expressions (Fox, 2002; Pourtois et al., 2013). Possibly, such an attentional grabbing allows a better evaluation of others’ intentions. Therefore, when threatening stimuli are used as Go-signals, they increase the RTs and the rate of omission errors (Mirabella, 2018; Mancini et al., 2020; Mirabella et al., 2022). Instead, when threatening stimuli act as No-go signals, they allow a more accurate inhibition, decreasing the rate of commission errors. This hypothesis should be tested in future studies assessing the allocation of visual attention during the Emotional and the Control (either the Gender or the Color) Go/No-go task using eye-tracking devices. A non-mutually exclusive explanation of current evidence comes from other studies suggesting that a positive mood increases flexibility, i.e., better processing of novel or unexpected stimuli, at the cost of increased distractibility, reducing goal maintenance (Dreisbach, 2006; Goschke and Bolte, 2014). To reconcile discrepant findings, Paul et al. (2021) proposed that these effects only occur when participants’ motivation is low. By contrast, when motivation is high, a positive mood has opposite effects, i.e., goal maintenance is increased stability, and attention is narrowed. Similarly, in all experimental settings, motivation is likely to be low in our experimental conditions. Thus, when task-relevant, the presentation of happy body images promotes participants’ distractibility, increasing the rate of commission errors.

### Limitations of the study

The current study has five main limitations. First, in the Color Discrimination Task, we instructed participants to look at the color of the t-shirts and not the whole picture. Thus, it could be argued that, in this case, the attention is on one part of the stimulus, which could explain why the emotional appraisal does not occur. However, on the one hand, often, to investigate the automatic effect of emotions on behavioral responses, the participant’s attention is directed to a completely different spatial location with respect to the emotional stimuli, e.g., to the colored frame of the image (e.g., see Sagaspe et al., 2011; Taylor et al., 2018). This is because the supporters of the idea of the automatic processing of emotional stimuli believe that it does not matter where the participant’s attention is. On the other hand, the upper part of the body is a part of a stimulus that conveys crucial hints about emotional images’ content (Dael et al., 2012; Kret et al., 2013). In fact, there is evidence that participants never pay attention to the lower part of the body to recognize emotions (Pollux et al., 2019). In addition, it has been demonstrated that during discrimination tasks, the upper part of the body seems to be a better marker than the lower one to recognize emotions (Reed et al., 2006; Arizpe et al., 2017; Reed et al., 2018). Therefore, we are in a more challenging situation than in other experimental designs studying the behavioral effect of emotional stimuli as the color cue is superimposed on the more relevant area in the picture for emotion recognition. In any case, future studies are needed to confirm our argumentation. Second, in the Emotional Discrimination task, we cannot assess whether the impact of emotional body expressions on inhibitory control is different from that induced by neutral body postures, as they are never used as No-go signals. However, we can compare the effect of neutral versus emotional body postures on inhibitory proficiency in the Color Discrimination task. In such an instance, i.e., when the emotional content of the stimuli is task-irrelevant, participants had a similar rate of commission errors for emotional and neutral expressions. Instead, in the Emotional Discrimination tasks, the rate of commission errors increased significantly for both happy and fearful body postures with respect to the Color Discrimination task, likely reflecting a greater difficulty of the former. Still, the rate of commission errors elicited by happy bodies in the Emotional Discrimination task is higher than that elicited by fearful bodies. Thus, we interpret such evidence as an indication that inhibitory control is modulated by the task relevance of stimuli’ emotional content. In our opinion, the design of the Emotional Discrimination task is the only one that allows studying the effect of two different task-relevant emotions for the same movements without informing participants about what emotions will be presented. Third, we used just two emotions (fear and happiness), and future studies should investigate the effect of the others, e.g., sadness and anger. This is very relevant as different emotions convey different signals and, thus, when task-relevant, should trigger different motor behaviors. For instance, while fear indicates the presence of a potential threat-inducing behaviors aimed to detect its source, anger conveys a direct threat toward the observer, eliciting defensive or attacking behaviors according to the context (Carver and Harmon-Jones, 2009). Fourth, we studied the effect on inhibitory control of facial (Mancini et al., 2022) and body emotional expressions separately. It would be of great interest to investigate the impact of pictures that show at the same time both features, as they would be more ecologically valid stimuli. In fact, it has been found that emotional body postures could influence the perception of facial expressions (Aviezer et al., 2012; Van den Stock and de Gelder, 2014). Fifth, in our study, we exploited two-dimensional static emotional pictures; however, those stimuli have a relative ecological value. A promising way to study the impact of emotional processing on motor control in settings resembling more real-life social situations is to employ virtual reality to build three-dimensional avatars. Such an approach will allow the creation of naturalistic events while maintaining a highly controlled experimental environment (Basbasse et al., 2022).

## Conclusion

We showed that emotional body postures impact inhibitory control only when task-relevant for the first time. This finding parallels our previous results on facial emotions (Mancini et al., 2022), indicating the generalizability of such a phenomenon. Previous findings concur to strengthen such a conclusion by showing that the impact of stimuli’ emotional content on motor behavior depends on the tasks’ goals in different i) aspects of motor control such as motor planning (Mirabella, 2018; Mancini et al., 2020), and inhibitory control (Mancini et al., 2022); ii) effectors [arm movements (Mirabella, 2018; Mancini et al., 2020, 2022), and forward gait initiation (Mirabella et al., 2022)], and iii) emotional stimuli (angry, happy and fearful faces and now on happy and fearful body postures). All in all, our research supports the notion that the effects of stimuli laden with emotional significance depend critically on their context-related evaluation, as theorized by the appraisal theories of emotions (Moors and Fischer, 2019; Scherer and Moors, 2019). By asserting this, we do not deny the possibility that behavioral reactions to emotional stimuli could be rapid and automatic in a situation of real danger for our survival. However, in most of our lives, we do not face such extreme events. Thus, emotional stimuli are usually appraised according to people’s goals. Relevantly, we do not deny that task-irrelevant stimuli cannot alter brain activity (Berkman et al., 2009; Sagaspe et al., 2011). However, such activations do not impact overt motor behaviors. Finally, our findings are restricted to healthy participants. Mental and neurological pathologies might profoundly influence emotion processing so that task-irrelevant emotional stimuli can induce motor reactions automatically. For instance, it has been shown that in patients with hemispatial neglect, images of fearful body postures automatically attract patients’ spatial attention even when presented on the neglected side (Tamietto et al., 2007). Future studies will have to assess the effect of task relevance in diseases affecting emotion processing, such as anxiety disorders and Parkinson’s disease.

## Data availability statement

The datasets presented in this study can be found in online repositories. The names of the repository/repositories and accession number(s) can be found at: https://osf.io/tykse/.

## Ethics statement

The studies involving human participants were reviewed and approved by Local ethical committee “ASST Spedali Civili” of Brescia, Italy (protocol number 4452). The patients/participants provided their written informed consent to participate in this study.

## Author contributions

MC, MM, and GM conceived the presented idea and planned the experiment. MM, CP, and EA carried out the experiments. MC, MM, CP, and GM performed the analysis. GM took the lead in writing the manuscript. All the authors interpreted and discussed the results. All authors provided critical feedback and helped to shape the research, analysis, and manuscript. All authors contributed to the article and approved the submitted version.

## Funding

This work was supported by the Antonio Meneghetti Award 2019 and by Erasmus+ project 610134-EPP-1-2019-1-JO -EPPKA2-CBHE-JP.

## Conflict of interest

The authors declare that the research was conducted in the absence of any commercial or financial relationships that could be construed as a potential conflict of interest.

## Publisher’s note

All claims expressed in this article are solely those of the authors and do not necessarily represent those of their affiliated organizations, or those of the publisher, the editors and the reviewers. Any product that may be evaluated in this article, or claim that may be made by its manufacturer, is not guaranteed or endorsed by the publisher.
